# Direct fluorogenic detection of palladium and platinum organometallic complexes with proteins and nucleic acids in polyacrylamide gels

**DOI:** 10.1038/s41598-020-69336-w

**Published:** 2020-07-23

**Authors:** Vladimir Pekarik, Marie Peskova, Jakub Duben, Marek Remes, Zbynek Heger

**Affiliations:** 1 Institute of Physiology, Faculty of Medicine, Masaryk University, 625 00 Brno, Czech Republic; 2 Central European Institute of Technology (CEITEC), Masaryk University, 625 00 Brno, Czech Republic; 3 Department of Chemistry and Biochemistry, Mendel University, Zemedelska 1, 613 00 Brno, Czech Republic; 4 Central European Institute of Technology (CEITEC), Brno University of Technology, 621 00 Brno, Czech Republic

**Keywords:** Biological techniques, Analytical biochemistry, Imaging

## Abstract

Allyl- and propargyl ethers of umbelliferone are sensitive probes for palladium and platinum, including anticancer compounds cisplatin, carboplatin and oxaliplatin, and effective for direct visualization of protein and DNA complexes with organometallic compounds in polyacrylamide gels allowing easy detection of interactions with analyzed protein or nucleic acid. Both probes can be used for fast evaluation of Pd/Pt binding to nanocarriers relevant in drug targeted therapy or specific clinically relevant target macromolecules.

## Introduction

Since the discovery of cisplatin as a potent anti-cancer compound, the general interest in therapeutic metallocomplexes dramatically increased. Identification of other platinum compounds with improved therapeutic properties further fueled the search for biologically active metallosubstances, which nowadays besides Pt and Pd, contain organocomplexes of gold, ruthenium^1^, rhodium and iridium^2^. Recent years have also seen a growing interest in palladium compounds that have therapeutic activity or whose catalytic activity can be used for prodrug activation in situ, in a therapeutic approach known as bioorthogonal therapy^3^.

Current trends in cancer therapies have shifted from systemic drug administration to targeted cancer drug delivery utilizing various types of nanocarriers. This raises a practical problem of detecting whether the therapeutic compound has been effectively encapsulated. Alternatively, metallodrugs might be directed against a specific protein (enzyme, regulatory molecule) and a facile technique is of utmost interest to detect such interactions. Methods used so far are mainly physio-chemical and require costly instrumentation available only to a limited number of laboratories. The techniques can also mostly detect only the presence of the metal ions but not their binding to a protein or nucleic acid. Binding of Pt and Pd complexes to DNA and proteins has been studied previously. In order to detect the formation of metal adducts with DNA spectrophotometric techniques detecting subtle changes in DNA absorption are used^4^. Binding of Pt complexes to protein can be analyzed by electrospray ionization – mass spectroscopy (ESI–MS) and has been used to detect binding of Pt-benzimidazole complexes to lysozyme^4^. This technique is precise but requires demanding samples preparation, special instrumentation, and is able to detect only stable coordination complexes between specific aminoacid and metal. In addition, the technique cannot be used to determine hydrophobic and other non-covalent or unstable coordination interactions. Pt coordination complexes that are very stable could be determined by ESI–MS but Pd complexes where the ligand exchange rate is much faster might be missed.

We are interested in encapsulation of Pd and Pt complexes into protein nanocarriers, specifically ferritins for targeted drug delivery^5^. Encapsulation of anticancer drugs, such as doxorubicin or ellipticine can be easily monitored due to inherent drugs fluorescence but there is no fast and simple technique to monitor metallocompound encapsulation.

That is why we have set to develop a technique that would permit direct visualization of such complexes with laboratory equipment commonly found in molecular biology labs using routine separation techniques such as polyacrylamide electrophoresis and detection system with standard laboratory transilluminator. The technique is not intended to analyze complex reaction mixes but to provide an insight whether a newly developed substances will interact with desired target macromolecule or evaluate whether the compound is effectively encapsulated in a given nanocarrier.

Majority of chemical sensors for the detection of noble metals utilizes their catalytic properties^6^ with Pd sensors often based on allyl or propargyl carbamates, carbonates, or ethers of phenolic fluorophores^6–10^. The fluorescent activation of the probes is based on the Tsuji-Trost allylic oxidative insertion mechanism^11^. The Pd^0^ oxidatively inserts into the allylic C–O bond of the non-fluorescent allylic/propargylic ether and the complex reacts with a nucleophile consequently releasing a fluorescent alcohol and an allylated or propargylated nucleophile. Many Pd complexes in a form of homogenous or heterogenus catalyst were used in cellular context^12–15^ to activate various fluorescent probes but the interaction with cellular proteins cannot be identified in this way.

Previously produced probes based on Pittsburgh Green^9,10^, dichlorofluorescein^16^, resorufin^8^, 4-hydroxynaphthalimide^17^ are sensitive and allow facile detection of Pd in quantitative assays. However, they frequently retain a relatively high level of fluorescence under UV making them excellent probes for spectrophotometric assays and microscopy but less effective for the use with an UV transilluminator. The requirement for UV excitability and the lack of visible fluorescence of the probe ruled out most of the common fluorophores except coumarin-based compounds. Coumarins are blue fluorochromes used in probes design or as enzymatic substrates since 1955^18^. Recently, a vinyl ether of umbelliferone (UF) was used as a reporter to monitor the traceless release of alcohols in cells after tetrazine mediated uncaging^19^. Two probes based on 7-hydroxy-4-(trifluoromethyl)coumarin were developed by Ding et al. containing allyl carbonate and propargyl ether moiety^20^. Some of these probes were used in vivo to detect Pd complexes in cells^21,22^. However, the binding partner cannot be identified in a cellular context.

Here, we present new probes responsive to Pd, Pt and other transition metals, based on umbelliferone and demonstrate their use to detect protein and DNA complexes of Pd and Pt organometallic compounds as well as the metal complexes encapsulated into ferritin nanocarrier.

## Results and discussion

### Design, synthesis and evaluation of propargyloxy- and allyloxyumbelliferone (UF) probe

**Figure 1:**
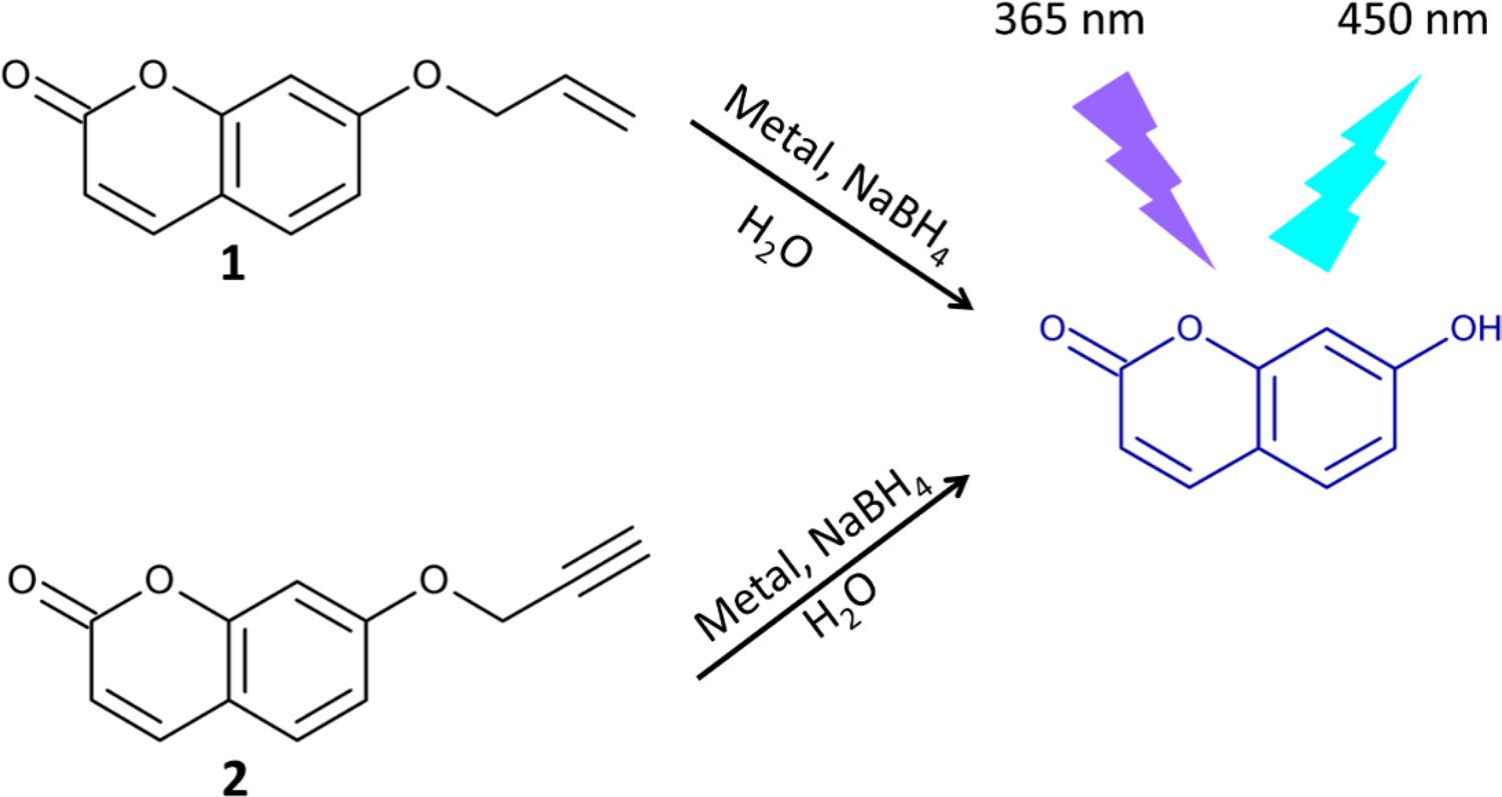
Mechanism of activation and detection of allyl- (**1**) and propargylumbelliferone ether (**2**).

The probe design is based on UF, a coumarin analogue, with a hydroxyl group that can be easily modified by the reaction with allyl or propargyl bromide. The UF was also chosen due to its good solubility in water and suitable fluorescence. The fluorochrome can be easily excited at 365 nm, a wavelength provided by transilluminators used to detect ethidium bromide stained DNA in agarose gels resulting in bright blue fluorescence (the mechanism of probes activation is schematized in Fig. 1). The UF deprotonation is prerequisite for its fluorescence. Attachment of allyl- or propargyl groups to the UF hydroxyls effectively blocks the fluorescence upon excitation with UV at 365 nm in alkaline medium (Fig. S3).

**Figure 2:**
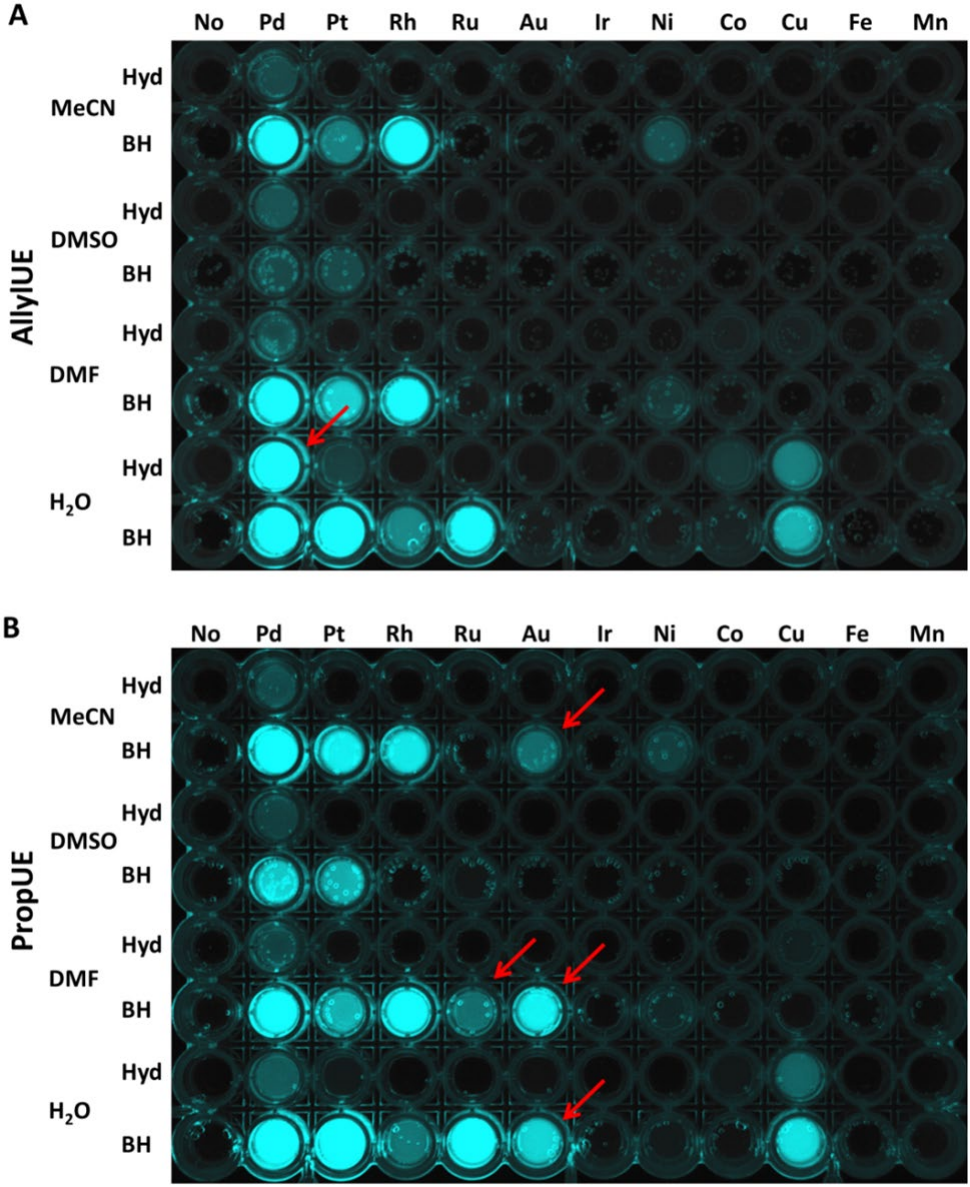
Activation of **1** (**A**) and **2** (**B**) by various metals (10 μM) in different solvents and reducing agents in a 96-well plate placed under UV transilluminator. Pd = Pd(OAc)_2_, Pt = K_2_PtCl_4_, Rh = RhCl_3_, Ru = RuCl_3_, Au = AuCl_3_, Ir = K_3_IrCl_6_, Ni = Ni(OAc)_2_, Co = CoCl_2_, Cu = CuSO_4_, Fe = FeCl_3_, Mn = MnCl_2_, Hyd = hydrazine hydrate (10 mM), BH = sodium borohydride (5 mM), MeCN = acetonitrile (20%), DMSO = dimethylsulfoxide (20%), DMF = dimethylformamide (20%). The total reaction volume was 200 µL with 25 µM **1** or **2**. The reaction time was 30 min. Red arrows highlight the differences between probes **1** and **2**.

We have tested several reactions conditions based on previously published results. These included sodium borohydride (NaBH_4_)^16^ and hydrazine (NH_2_NH_2_)^7^ as the reducing agents in 20% acetonitrile (MeCN), dimethyl sulfoxide (DMSO), dimethylformamide (DMF), and in pure water. The use of triphenylphosphine (TPP)^9^ resulted in fluorescent artefacts and was therefore excluded from further experiments. Figure 2 demonstratesthe effects of various metal ions on efficiency of the probes activation .

The results demonstrate that both probes are efficiently activated by Pd, Pt, Ru, Rh, and Cu. In the case of **2**, a very strong signal appears also in response to the presence of Au. Reactivity of both probes was outstanding in pure water in the presence of BH compared to mixed solvents and Hyd as a reducing agent. Quantitative data are summarized in Fig. S4. These results indicate that the sensitivity of other allyl/propargyl ether probes synthesized by other groups could be further modified by changing the reaction conditions. These results can also aid in choosing appropriate deprotection conditions of organic molecules where allyl ether is used as a protecting group during chemical synthesis.

Since Pd is commonly used as a catalyst in the preparation of pharmaceutical substances and Pd contamination is of great health concerns, we were curious what would be the detection limit for Pd used for quantitative Pd determination.

The reaction was carried out in 20% ACN in order to improve the solubility of potential analytes.

We found that the detection limit for Pd(OAc)_2_ at an increased gain (60) is ~ 20 nM. The saturation is reached at Pd concentrations ~ 4 µM. Importantly, by a naked eye, Pd can be determined in a 96-well plate on a transilluminator in concentrations exceeding 150 nM (Fig S5).

### Detection of organometallic compounds

**Figure 3:**
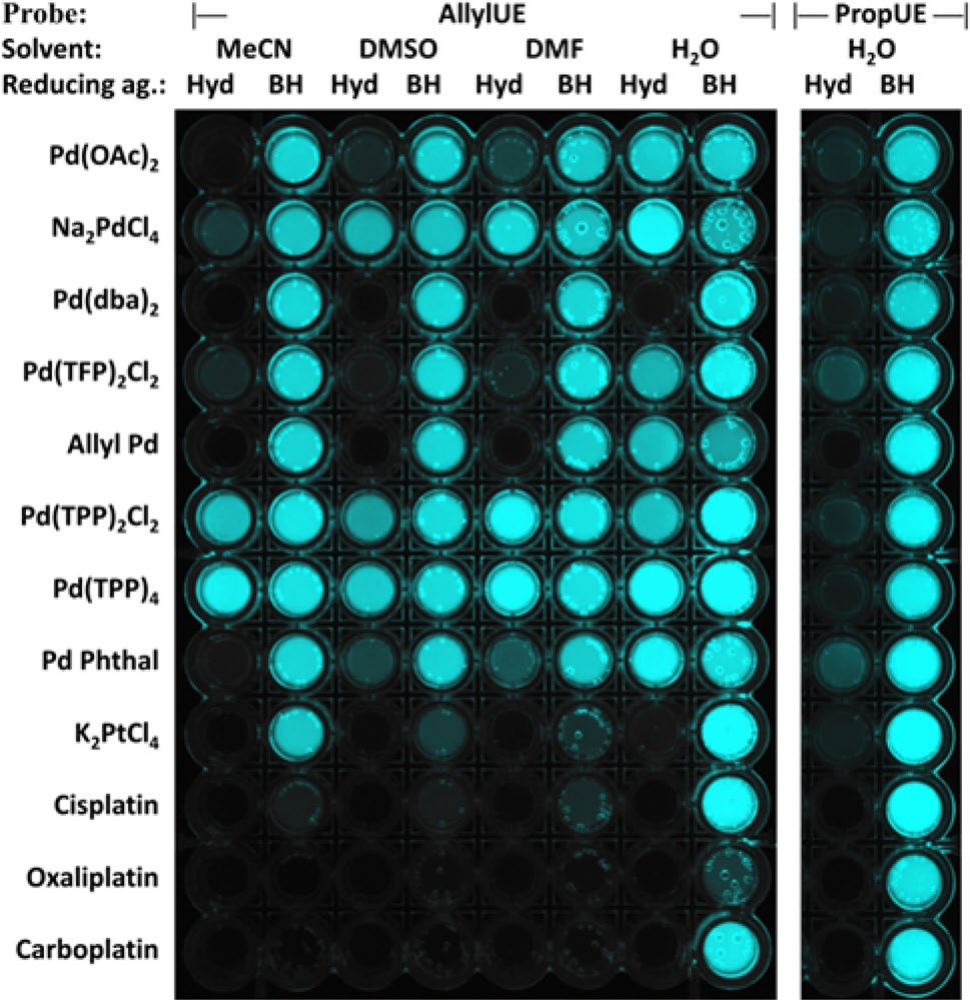
Activation of AllylUE (**1**) and PropUE (**2**) probes in presence of Pd or Pt complexes (10 μM) in various solvents and reducing agents in a 96-well plate placed on a UV transilluminator. Pd(OAc)_2_ = palladium(II) acetate, Na_2_PdCl_4_ = sodium tetrachloropalladate(II), Pd(dba)_2_ = bis(dibenzylideneacetone)palladium(0), Pd(TFP)_2_Cl_2_ = bis(tri(2-furyl)phosphine)palladium(II) dichloride, AllylPd = Allylpalladium(II) chloride dimer, Pd(TPP)_2_Cl_2_ = bis(triphenylphosphine)palladium(II) dichloride, Pd(TPP)_4_ = tetrakis(triphenylphosphine)palladium(0), Pd Phthalocyanine = phthalocyanine palladium(II), K_2_PtCl_4_ = potassium tetrachloroplatinate(II), Hyd = hydrazine hydrate (10 mM), BH = sodium borohydride (5 mM), MeCN (acetonitrile (20%), DMSO = dimethylsulfoxide (20%), DMF = dimethylformamide (20%). The total reaction volume was 200 µL with 25 µM AllylUE or PropUE. The reaction time is 30 min.

Due to the strong response of both probes to inorganic metal ions, we were curious whether **1** and **2** can detect organometallic Pd and Pt compounds. The results show that all tested Pd complexes, and surprisingly also anticancer drugs cisplatin and carboplatin induce a strong increase in fluorescence (Fig. 3) of the assay carried out in water with BH. The inefficiency of Hyd to activate the probe in presence of Pt compounds could be explained by the inability of Hyd to reduce Pt^2+^ into Pt^0^. These results are exciting because only a few fluorescent sensors, such as rhodamine triazole^23^, diethyldithiocarbamate-rhodamine B^24^, resorufin allyl ether^8^, and Pittsburgh green allyl ether^10^ are sensitive to Pt compounds. All published probes respond differently to various Pt compounds; however, the sensitivity of only a few of them has been tested against anticancer complexes. Taken together, our results demonstrate that **1** and **2** are useful for the detection of some platinum anticancer compounds. The lack of activation of the probe with cisplatin, oxaliplatin, and carboplatin in presence of organic solvents can be possibly explained by the formation of stable complexes with DMSO or acetonitrile. Pohorilets et al.^25^ have analysed mechanism of Tsuji-Trost reaction in detail and has shown that excess of DMSO represses Pd mediated activation of allyl Pittsburg green.

### Detection of Pd and Pt compounds in biological fluids

**Figure 4:**
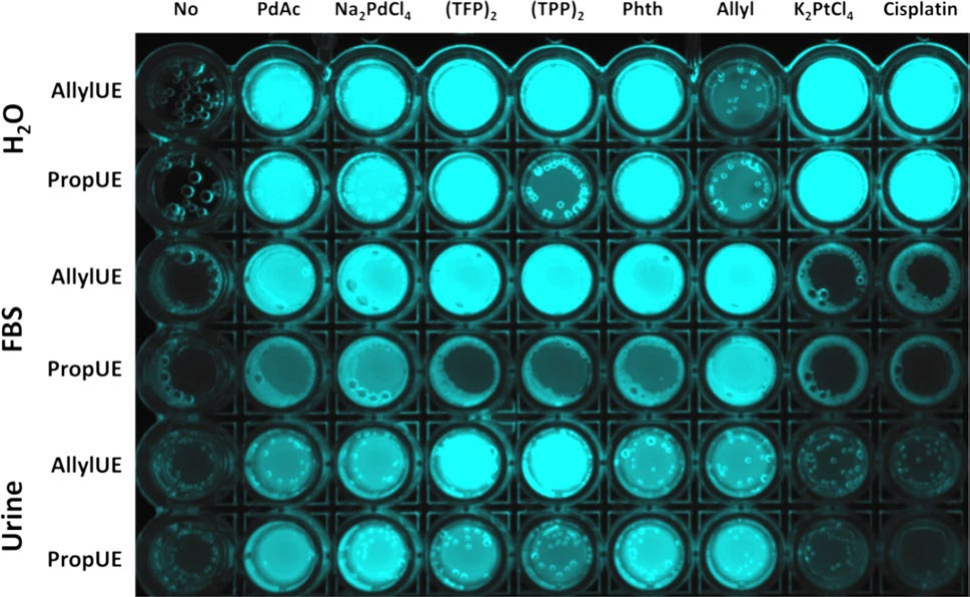
Activation of AllylUE (**1**) and PropUE (**2**) probes by Pd catalysts and Pt drugs in fetal bovine serum (FBS) and urine. Pd catalysts and Pt drugs were mixed with 50 μL of FBS or urine in 96-well plate, incubated for 30 min at 25 °C, and 150 μL of reaction mixture with AllylUE or PropUE and BH in water was added with final concentrations of 5 μM catalyst, 25 μM probe, and 5 mM BH and incubated for 1 h at 23 °C. The plate was visualized on a UV transilluminator. PdAc = palladium(II) acetate, (TFP)_2_ = bis(tri(2-furyl)phosphine)palladium(II) dichloride, (TPP)_2_ = bis(triphenylphosphine)palladium(II) dichloride, Phth = phthalocyanine palladium(II), Allyl = Allylpalladium(II) chloride dimer. In all samples can be seen bubbles of H_2_ produced by decomposition of BH.

Clinical use of metallodrugs prompted us to test whether UF probes can be used for the detection of Pd and Pt compounds in biological fluids (serum, urine). We have reacted **1** and **2** with Pd or Pt compounds pre-incubated with urine or fetal bovine serum (Fig. 4). All Pd compounds can be easily detected, while Pt compounds do not activate any probe in the presence of serum or urine, precluding the possibility to detect organoplatinum compounds in blood or urine of patients undergoing chemotherapy. Nevertheless, detection of drugs should not be affected by infusion solutions, and can be thus possibly used for sensing of cytostatic contaminations in the hospital environment.

### Direct visualisation of the interaction between Pd metallodrugs and proteins

The previous experiment confirmed that both probes are sensitive to Pd and Pt compounds. With the optimized reaction conditions, we wanted to see whether Pd/Pt complexes with biomolecules can be visualized in native polyacrylamige gels (PAG). As a model protein, we have used recombinant ferritin originating from hyperthermophilic archaea *Pyrococcus furiosus* (Pfu)^26^, which is a promising drug nanocarrier. For comparison, we have used bovine serum albumin (BSA), which is a major plasma carrier protein important for binding and transport of many pharmacologically active substances^27^.

**Figure 5:**
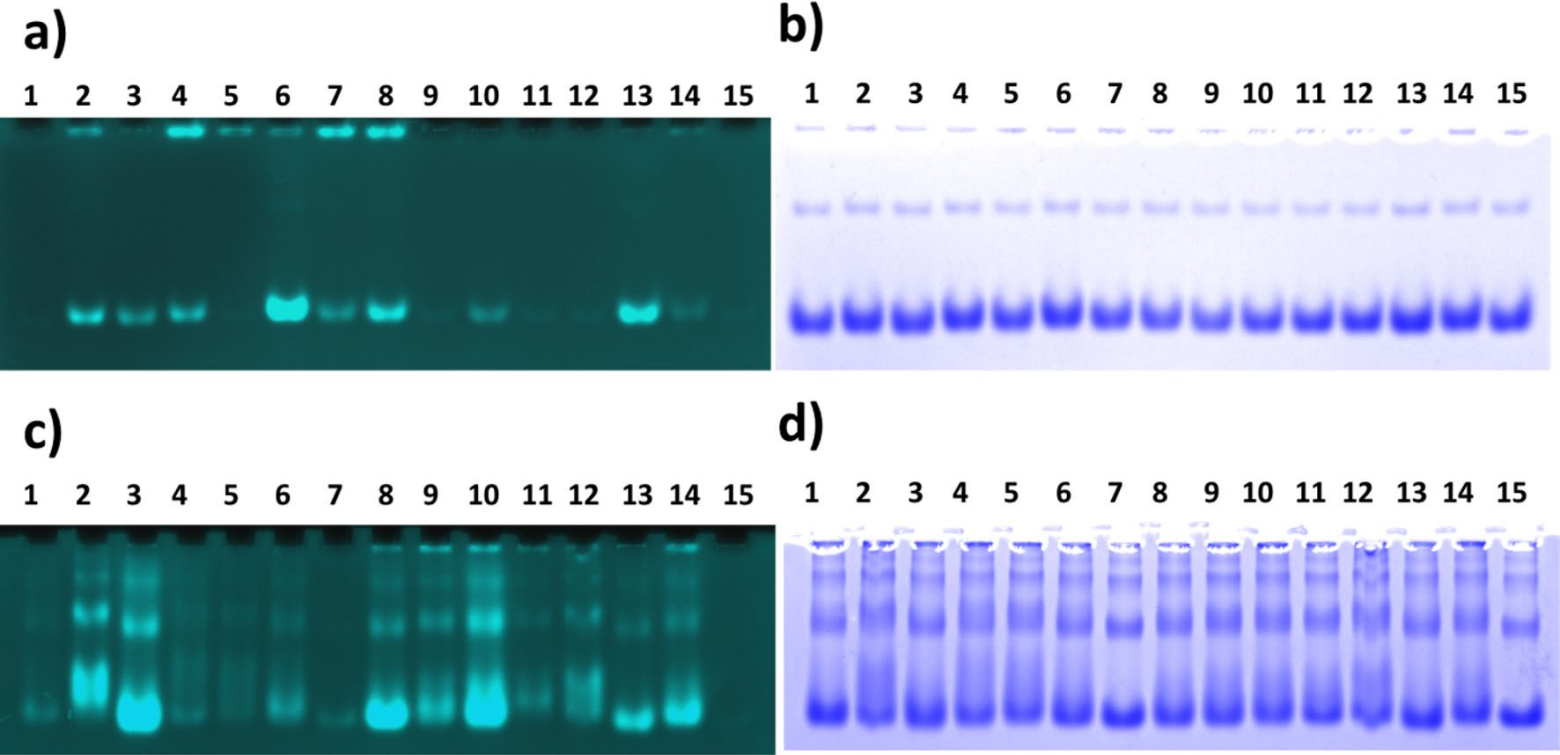
Activation of AllylUE (**1**) in native PAG by Pd complexes with Pfu ferritin (**a**, **b**) and BSA (**c**, **d**). The complex was formed by incubation of 20 µg of protein with 100 µM catalyst for 12 h. The complexes were resolved in 5% native PAG in Tris–glycine buffer. The Pd catalyst was visualised with AllylUE (*left*) and the same gel was stained for detection of protein with Coomassie blue (*right*). The used catalysts were: 1—None, 2—Pd(OAc)_2_, 3—Na_2_PdCl_4_, 4—Pd(TFP)_2_Cl_2_, 5—Pd(TPP)_2_Cl_2_, 6—Pd Phthalocyanine, 7—Pd-BBDPPE, 8—Pd(hfacac), 9—AllylPd, 10—Pd-Spermine, 11—Pd(bipy)Cl_2_, 12—PdPhen(OAc)_2_, 13—Pd(TNFB)Cl_2_, 14 – Pd_2_(dipic)_2_(TPP)_2_, 15—Pd^0^(Phen)_2_.

Both proteins were incubated with a series of Pd compounds and separated on native PAG. The Pd complexes were detected by a direct application of NaBH_4_ solution to the surface of gels previously soaked with the probe **1** and placed on top of a common laboratory UV transilluminator (Fig. 5).In the same gel, the proteins can be detected by subsequent staining with Coomassie blue. The colocalisation of signals confirms the interaction between the studied organometallic compound and given macromolecule.

The images of PAGs shown in Fig. 5 revealed differences in interactions between Pd-complexes and either *P. furiosus* ferritin or BSA. The results show a very high affinity of Pfu ferritin for Pd-phthalocyanine, Pd(hfacac), Pd(TNFB)Cl_2_ while other catalysts bind moderately or not at all. On the other hand, BSA binds strongly inorganic Pd (Pd(OAc)_2_ and Na_2_PdCl_4_), Pd(hfacac), and Pd-spermine complex while other catalysts interact with BSA only moderately. The results demonstrate that our probes enable facile and sensitive detection of binding of Pd complexes to proteins and show clear distinctions in complex formation between various proteins with evaluated Pd compounds. It is worth to mention that the signal development might be accompanied by formation of H_2_ bubbles underneath the gel and in order to acquire good images these bubbles should be pushed away by rolling a pipette over the gel.

### Direct visualization of interactions between Pd metallodrugs and nucleic acids

**Figure 6:**
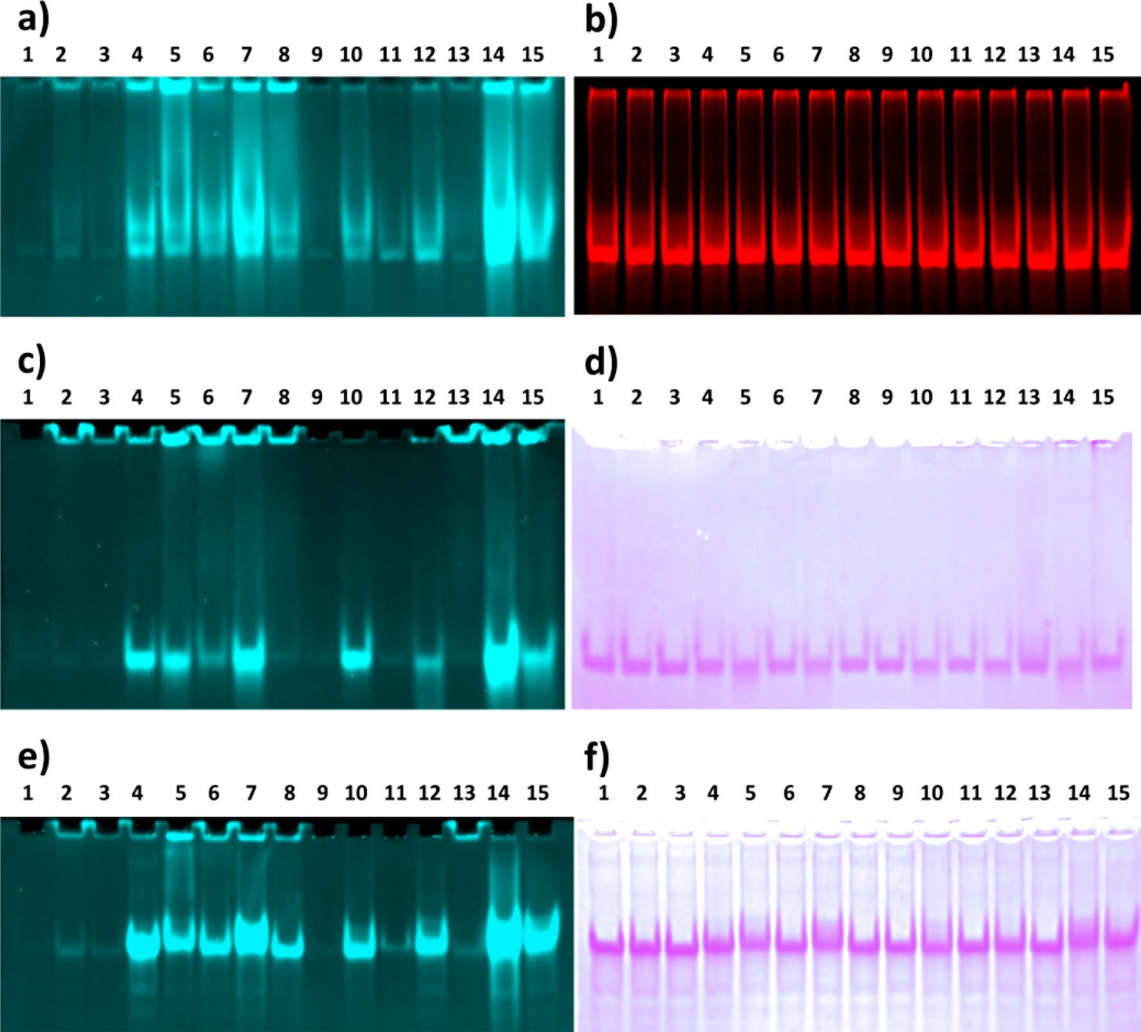
Activation of AllylUE (**1**) (**a**, **c**, **e**) in PAG gels by Pd complexes with dsDNA (**a**, **b**), qDNA (**c**, **d**), and ssDNA (**e**, **f**). The gels after detection of AllylUE activation were stained with ethidium bromide (**b**) or crystal violet (**d**, **f**) to visualise the DNA. The complex was formed by incubation of 10 µM oligonucleotide with 100 µM catalyst. The complexes were resolved in 16% PAG in Tris–Borate buffer. The used catalysts were: 1—None, 2—Pd(OAc)_2_, 3—Na_2_PdCl_4_, 4—Pd(TFP)_2_Cl_2_, 5—Pd(TPP)_2_Cl_2_, 6—Pd Phthalocyanine, 7—Pd-BBDPPE, 8—Pd(hfacac), 9—AllylPd, 10—Pd-Spermine, 11—Pd(bipy)Cl_2_, 12—PdPhen(OAc)_2_, 13—Pd(TNFB)Cl_2_, 14—Pd_2_(dipic)_2_(TPP)_2_, 15 Pd^0^(Phen)_2_.

Since some clinically used metallocomplexes are specifically designed to interact with cellular DNA we have tested whether we can use the established technique to detect the interaction of Pd complexes with DNA, specifically double-stranded (dsDNA) and single-stranded DNA (ssDNA). For comparison, we utilized DNA oligonucleotide Ag45 forming G-quadruplexes tertiary structure (qDNA)^28^. G-qadruplexes are structural motives present in telomeric regions of chromosomes that became target of new anticancer interventions. The Pd complexes were detected with probe **1** and DNA in the same gel was detected by subsequent staining with ethidium bromide (dsDNA) or crystal violet (ssDNA, qDNA) (Fig. 6). The results confirm an effective binding of some Pd complexes to DNA, though we have seen no substantial difference between affinities to various structural types. Inorganic salts, allylPd, and Pd(TNFB)Cl_2_ bind very poorly to any DNA. Pd(hfacac) interacts with dsDNA and ssDNA but the binding to qDNA is compromised. Noteworthy, Pd^0^(Phen)_2_, which did not interact with PfuFt or BSA has a strong affinity for all types of DNA. We have also tested whether complexes of Pt and Ru with DNA can be detected by the proposed technique (Fig. S6). Regretably, we have not seen UPE activation by any tested comlex. We assume that very high concentration of acrylamide (16%) used to separate the small oligonucleotide fragments might be responsible because the Pt complexes with proteins separated in 5% acrylamide gels can be detected without problems. Neither borate present in the DNA separation gel nor DNA itself interfere with the reaction (data not shown).

The technique of detecting palladium, and possibly adapted for other metals, in complexes with nucleic acids can find its use also in synthetic biology, specifically in design of new artificial metal base pairs. In a metal-mediated base pair, the hydrogen bonds of a canonical base pair are formally replaced by coordinate bonds to one or more metal ions located in the interior of the duplex^29^. The promissing strategy is the replacement of nucleotides with imidazole derivatives, such as 1-(2-deoxy-β-D-ribofuranosyl) imidazole-4-carboxylate nucleoside triphosphate (dImCTP)^30^. The presence of silver ions supports the unusual coordination pattern. Such synthetic nucleic acids can find its use in nanotechnology, supramolecular coordination chemistry etc. Of interest might be a design of new atomic metal arrays assembled in artificial DNA. This concept has been described for copper coordination array coordinated in hydroxypyridone nucleobases^31^. Replacement of copper with palladium or other metals might lead to the development of effective catalyst with potential applications in bioorthogonal chemistry.

### Direct visualization of interactions between anticancer platinum compounds and ferritin nanocarriers

Unfortunately, we found that Pt complexes with proteins or DNA cannot be detected in PAG. In order to address this issue, we have tested individual components of PAG for the reaction inhibition and found that the inhibitory compound is acrylamide itself. We hypothesized that acrylamide that remains incompletely polymerized in the gel, does interact with Pt and suppresses its catalytic activity. Moreover, we hypothesized that supplementing the system with another metal ion that would compete with Pt for binding to acrylamide might liberate Pt and revive its catalytic activity and permit the probe activation.

**Figure 7:**
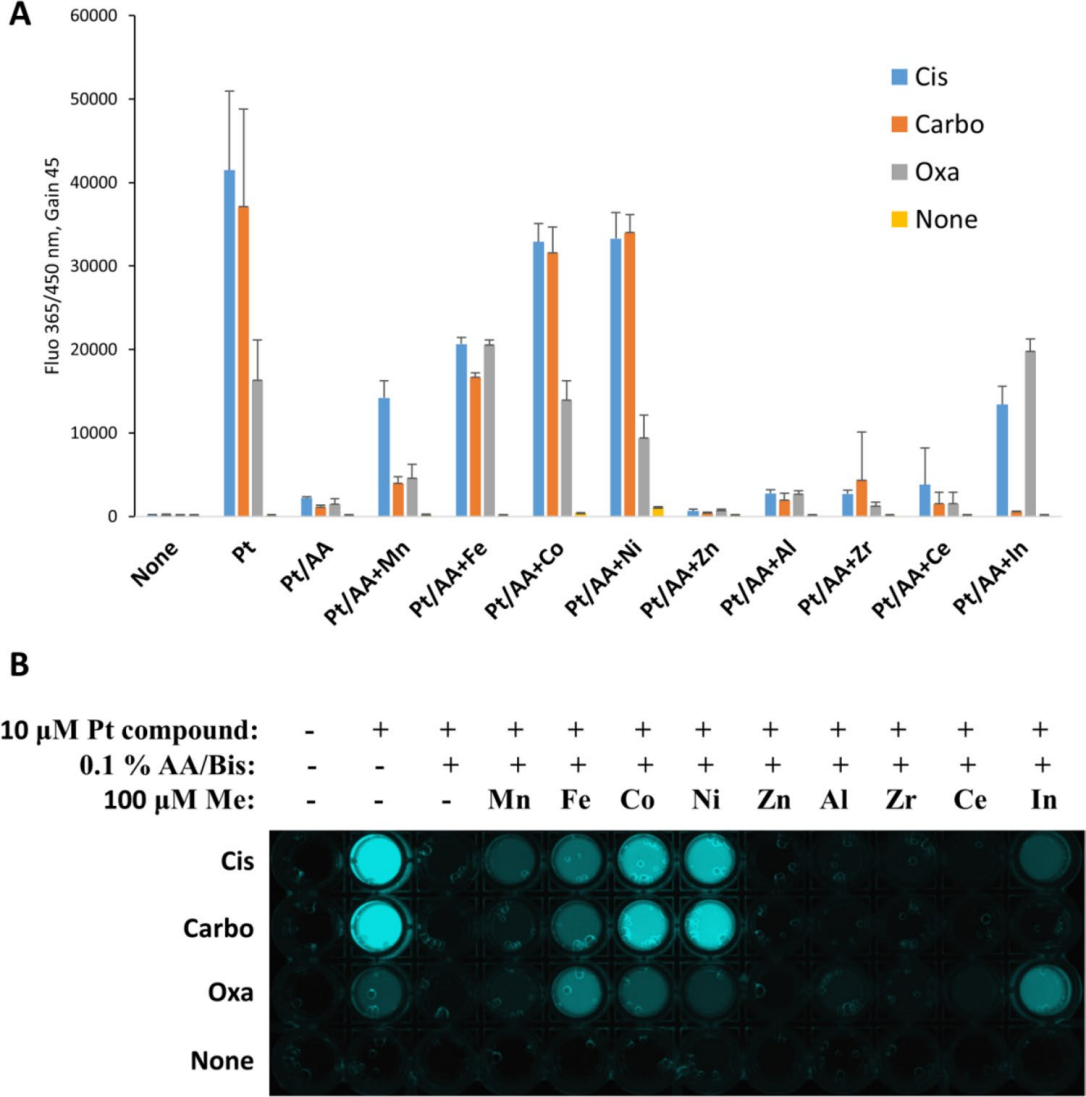
Restoration of catalytic activity of acrylamide-inhibited Pt compounds. Cisplatin (Cis), carboplatin (Carbo), and oxaliplatin (Oxa) were incubated with 0.1% acrylamide (Pt/AA) for 10 min. Then, competing metal was added and after 20 min incubation, the reaction mix containing **2** and BH was added. In order to exclude the probe activation by the competing metal, control samples without Pt compounds (yellow bars) were included. Fluorescence was measured after 60 min incubation. Results are the average of triplicate measurements (**A**). (**B**) The appearance of the 96-well plate under transilluminator. Mn = MnCl_2_, Fe = FeCl_2_, Co = CoCl_2_, Ni = Ni(OAc)_2_, Al = AlCl_3_, Zr = ZrOCl_2_,Ce = CeCl_3_, In = In(OAc)_3_.

In order to prove the hypothesis, we have mixed cisplatin, carboplatin, and oxaliplatin with acrylamide and other metal ions (that do not activate the probe itself) in 10 molar excess and carried out the deprotection assay. Surprisingly, we found that some catalytic activity is restored after addition of Co^2+^ or Ni^2+^, and to a lesser extent Fe^2+^ (Fig. 7) for all tested cytostatics. The sensitivity of the probe to cisplatin is also partially restored by Mn^2+^, and In^3+^, which also reactivates oxaliplatin. The results also point toward a new direction in metal-catalyzed reactions, in which the catalyst activity can be enhanced by supplementing the system with co-catalyst metal.

Next, we incubated Pt compounds with PfuFt and human H-chain ferritin (FtH), which is another perspective drug nanocarrier^32^. The samples were separated in native PAG and one gel was stained with **2** by the standard technique while the second gel was preincubated in 1 mM CoCl_2_ in 50 mM HCl before staining. The HCl was included to prevent precipitation of Co(OH)_2_ in alkaline pH of the Tris-buffered gel.

**Figure 8:**
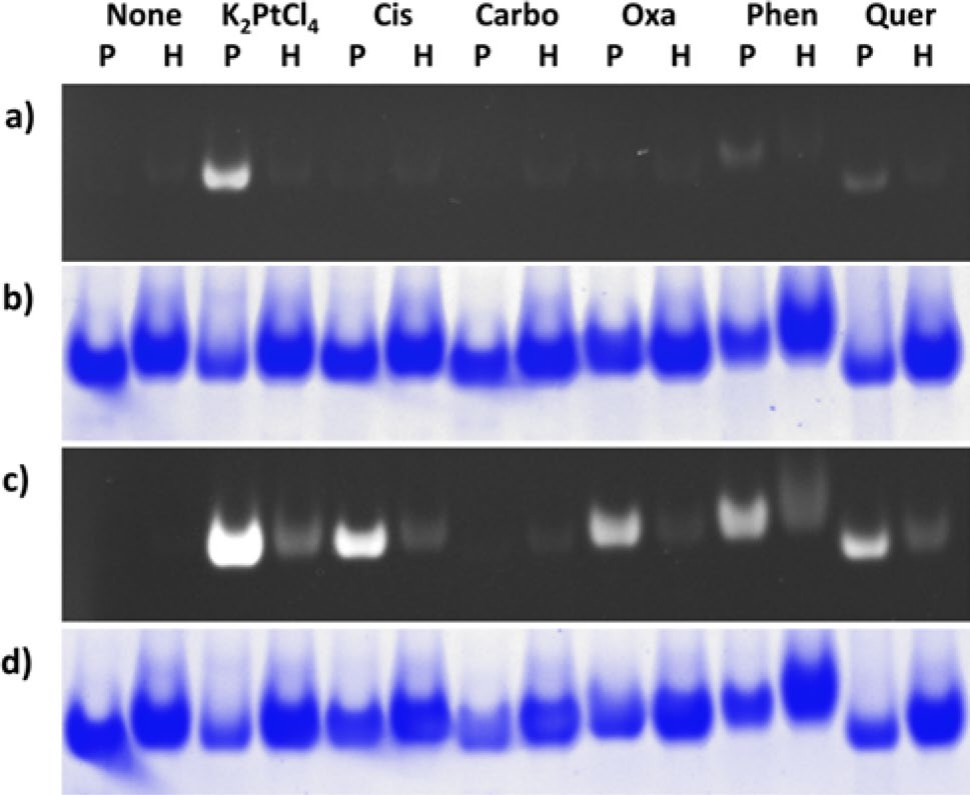
Detection of Pt complexes with PfuFt (P) and human FtH (H) ferritins in native PAG without (**a**, **b**) and with Co^2+^ treatment (**c**, **d**) with **2**. Used compounds were: None, K_2_PtCl_4_, cisplatin (Cis), carboplatin (Carbo), oxaliplatin (Oxa), Pt-phenanthroline (Phen), and Pt-quercetin (Quer). Gels were stained with **2** (**a**, **c**) followed by staining with Coomassie blue (**b,** **d**) to detect proteins in the same gel.

The results shown in Fig. 8 clearly validate the improvement of sensitivity toward Pt compounds upon Co^2+^-enhanced staining. The results also demonstrate the first practical use of the technique allowing identification of the most appropriate nanocarrier for a specific compound. In this case, PfuFt is a superior carrier for the majority of Pt compounds with the exception of carboplatin that poorly interacts with both proteins. Previously, ferritins have been successfully used for cisplatin encapsulation. It was found that FtH is able to bind 5 molecules of cisplatin in each chain^33^, while horse spleen apoferritin binds only 1 cisplatin molecule^34^. From the results in Fig. 8, it is clear that PfuFt entraps a substantially larger quantity of therapeutic compound then FtH and might be therefore considered as a more effective carrier for nanomedical purposes.

## Conclusions

In conclusion, we have developed UV excitable blue fluorescent probes for the quantitative detection of ions and organometallic complexes of several transition metals. We demonstrate that the reaction in water with BH as a reducing agent provides superior results compared to conditions previously published. To the best of our knowledge, this is the first report, describing the methodology for the direct visualization of binding of Pd and Pt compounds to proteins or DNA. The catalytic activity of Pt compounds is suppressed by acrylamide, and potentially by other alkenes, but the inhibition can be reversed by Co^2+^ or Ni^2+^ treatment of the gel. Due to the growing interest in the development of new Pd and Pt anticancer compounds or possible applications of a Pd catalyst in bioorthogonal therapy, the identification of specific interactions with proteins, including serum transport proteins, is of crucial importance and the presented technique can greatly facilitate the task. The results of biochemical assays indicate that the technique can be further extended to the detection of protein and DNA complexes with organometallic compounds of Au, Rh, and Ru.

## Materials and methods

### Probe activation

The experiments were carried out in acrylic UV transparent 96-well plates (Corning, New York, NY, USA). Because the majority of the catalysts are dissolved in DMF, which dissolves the acrylic plates, 50 μL of 20% solvent was pipetted to the wells first. Then, 2 μL of a solution of 1 mM catalyst in DMF was added, and only then 150 μL of a master mix consisting of 20% solvent, Allyl- (**1**) ^35^ or PropUE (**2**) and reducing agents were added. The final concentrations were 25 μM for the probe, 10 μM for the catalyst, 5 mM for NaBH_4_, and 10 mM for hydrazine, unless specified otherwise. The plate was incubated for 20–60 min at ambient temperature. Fluorescence was measured with excitation at 365 nm and emission at 450 nm with various gains. All reactions were conducted in tri- or quadruplicates.

### Detection of Pd complexes in PAG

After electrophoretic separation, the apparatus was disassembled and the gel placed into a dish with 10 mL of 200 µM probe in water. With gentle agitation, the probe was allowed to soak into the gel for at least 30 min. The gel was briefly rinsed with distilled water and placed onto the laboratory UV transilluminator and excess of liquid was removed. Because some proteins have strong autofluorescence, a few images with different exposure times were collected to account for an autofluorescent signal. Then, 2 mL of freshly prepared 10 mM NaBH_4_ and used within 5 min from dissolving were poured to the surface of the gel and evenly spread with a plastic pipetting tip. The images at different exposure times were collected at various intervals within 5 or 6 min after the borohydride solution was applied. Sometimes, usually after 3 min, hydrogen bubbles started forming underneath the gel and had to be manually expelled to prevent interference with images acquisition. The procedure is similar to previously published detection of Pd nanoparticles in acrylamide gels^36^.

### Restoring catalytic activity of acrylamide-inhibited Pt compounds with metal ions

2 μL of 1 mM cisplatin, carboplatin and oxaliplatin with 50 μL of 0.1% acrylamide/bisacrylamide (37.5:1) were mixed in 96-well plate and incubated for 10 min at ambient temperature. Then, 20 μL of 1 mM tested metal solutions were added. After 30 min incubation, PropUE and BH mix (130 μL) was added with final probe concentration 50 μM and BH 5 mM, and the fluorescence was measured after 1 h incubation at ambient temperature.

### Cobalt-enhanced detection of Pt compounds in PAG

The sample preparation and electrophoresis were carried out as described above. After electrophoresis, the gel was soaked for 30 min in 20 mL of 1 mM CoCl_2_ in 50 mM HCl. The HCl was included to neutralized Tris pH 8.8 present in the native PA gel that would cause precipitation of insoluble Co(OH)_2_. The gel was briefly washed in water and soaked for 30 min in 100 μM solution of PropUE in 100 mM Tris.HCl pH 8.8. The Tris was included to restore slightly alkaline pH necessary for depropargylation reaction and for fluorescence activation of released UF.

## Supplementary information

Supplementary Information.
