# Supplementary material for: Direct fluorogenic detection of palladium and platinum organometallic complexes with proteins and nucleic acids in polyacrylamide gels
Source: Sci Rep. 2020 Jul 23;10:12344. doi: 10.1038/s41598-020-69336-w (PMC7378192; doi:10.1038/s41598-020-69336-w)
Supplement: Supplementary file 1 — Supplementary Information. [file 41598_2020_69336_MOESM1_ESM.pdf]

SUPPLEMENTARY INFORMATION

Direct Fluorogenic Detection of Palladium and Platinum Organometallic Complexes with Proteins and Nucleic Acids in Polyacrylamide Gels

Vladimir Pekarik[1,2]*, Marie Peskova[2], Jakub Duben[1], Marek Remes[3,4], Zbynek Heger[3,4]

*E-mail: pekarikv@mail.muni.cz

Palladium complexes abbreviations:
$Pd(OAc)_2$ - palladium(II) acetate
Na_2PdCl_4 - sodium tetrachloropalladate(II)
$Pd(TFP)_2Cl_2$ - bis(trifurylphosphine)palladium(II) dichloride
$Pd(TPP)_2Cl_2$ - bis(triphenylphosphine)palladium(II) dichloride
Pd Phtal - palladium phthalocyanine
Pd-BBDPPE - bis[1,2-bis(diphenylphosphino)ethane]palladium(0)
$Pd(hfacac)_2$ - palladium(II) hexafluoroacetylacetonate
AllylPd – allylpalladium(II) chloride dimer
$Pd(bipy)Cl_2$ – (2,2′-bipyridine)dichloropalladium(II)
$PdPhen(OAc)_2$ - palladium(II) phenanthroline diacetate
$Pd(TNFB)Cl_2$ - palladium(II) 4,4,4-Trifluoro-1-(2-naphthyl)-1,3-butanedione dichloride
$Pd(dipic)(TPP)_2$ - palladium(II) dipicolinic acid bis(triphenylphosphine)
$Pd^0(Phen)_2$ - bis(1,10-phenanthroline) palladium(0)

Material and Methods

Materials and apparatus

Organic solvents were purchased from Penta (Czech Republic). All other chemicals were from Sigma-Aldrich (St. Louis, MO, USA) unless specified otherwise, and were used without further purification. DNA oligonucleotides were synthesized by Sigma-Aldrich.

Synthesis of 1

To a solution of umbelliferone (810 mg, 5 mmol, 1 eq) in dimethylformamide (DMF, 10 mL), K_2CO_3 (2.42 g, 17.5 mmol, 3.5 eq) was added and the mixture was stirred at room temperature for 15 min. Allyl bromide (4.33 mL, 50 mmol, 10 eq) was then added. After stirring at room temperature for 30 min, the resulting mixture was heated to 70 °C for 5 h. The mixture was diluted with water (100 mL) and allowed to incubate at 25 °C for 30 min. The reaction mixture was then centrifuged for 5 min at 5 000 rpm and the aqueous phase was discarded. The remaining pellet was dissolved in 10 mL of ethyl acetate, and extracted with 1 M $NaHCO_3$ and brine. The solvent was removed under reduced pressure and the crude solid product was extracted 5× with 1 mL of MeOH, air dried yielding 407 mg of white solid corresponding to 40% yield. ^1H NMR (700 MHz, $CDCl_3$) δ 7.60 (d, J = 9.5 Hz, 1H), 7.33 (d, J = 8.6 Hz, 1H), 6.81 (dd, J = 8.6, 2.4 Hz, 1H), 6.75 (d, J = 2.5 Hz, 1H), 6.19 (d, J = 9.5 Hz, 1H), 6.00 (ddt, J = 17.3, 10.6, 5.3 Hz, 1H), 5.40 (ddd, J = 17.3, 3.0, 1.6 Hz, 1H), 5.29 (ddd, J = 10.6, 2.7, 1.3 Hz, 1H), 4.61 – 4.50 (m, 2H) ppm (**Fig S1**). Analytical data correspond to the literature. The compound **1** was also synthesized by an alternative procedure.

Alternative synthesis of 1.

Procedure 2. UF (1.73 g, 10.6 mmol) was dissolved in 50 mL of acetone and then potassium carbonate (2.07 g, 15 mmol) was added. Subsequently, allyl bromide (2.16 mL, 25 mmol) was added dropwise under stirring. The reaction suspension was stirred and refluxed at 70 °C for 12 hours. The resulting product was isolated after filtration of the reaction mixture and its evaporation under reduced pressure. The final product was purified by flash chromatography on silica gel (mobile phase c-hexane/ethyl-acetate 5:1) to yield 1.368 g of white solid corresponding to 64% yield based on UF. The procedure is based on the work of Kofoed.[1]

Synthesis of 2

To a solution of UF (405 mg, 2.5 mmol) in DMF (10 mL), K_2CO_3 (1.21 g, 8.75 mmol, 2.8 eq) and tetrabutylammonium bromide (40 mg, 0.125 mmol, 0.05 eq) were added, and the mixture was stirred at room temperature for 15 min. Then, propargyl bromide (80% in toluene, 1.125 mL, 6.3 mmol, 2.5 eq) was added. After stirring at room temperature for 30 min, the resulting mixture was heated to 60 °C for 7 h. The mixture was diluted with ethyl acetate (10 mL) and extracted with 1M Na_2CO_3. EtOAc layer was transferred to a new tube and the aqueous layer was extracted 3× with 5 mL of EtOAc. Combined organic layers were washed with brine and dried over $MgSO_4$. The product was crystalized from the organic phase and the crude solid product was extracted 5× with 0.5 mL of MeOH and air dried yielding 140 mg of white solid corresponding to 28% yield. ^{1}H NMR (700 MHz, CDCl3) δ 7.65 (d, J=9.4 Hz, 1H), 7.41 (d, J=8.7 Hz, 1H), 6.95 (d, J=2.6 Hz, 1H), 6.92 (dd, J=2.4, 8.5 Hz, 1H), 6.29 (d, J=9.5 Hz, 1H), 4.77 (d, J=2.4 Hz, 2H), 2.58 (t, J=2.4 Hz, 1H) ppm (**Fig S2**).

Synthesis of bis[tri(2-furyl)phosphine]palladium(II) dichloride ($PdCl_2(TFP)_2$).

$PdCl_2(TFP)_2$ was prepared according to the published procedure,[2] bis(acetonitrile)dichloropalladium(II) (100 mg, 0.39 mmol, 1.0 eq) was dissolved in dry MeCN (15 mL). Then a solution of tri(2-furyl)phosphine (TFP, 179 mg, 0.77 mmol, 2.0 eq) in dry MeCN (2 mL) was added. The reaction mixture was allowed to stir at ambient temperature for 16 hours. Yellow precipitate was collected, washed with water (2 x 10 mL), and dried under vacuum (73 mg, 30% yield). ^{1}H NMR (701 MHz, DMSO) δ 8.12-8.00 (m, 0.3H), 7.92 (s, 1H), 7.25-7.10 (m, 0.3H), 6.93 (s, 1H), 6.75-6.65 (m, 0.3H), 6.58 (s, 1H) (In agreement with published data).

Synthesis of palladium(II) phthalocyanine.

Synthesis of Pd Pthal was accomplished according to the published procedure.[3] A mixture of phthalonitrile (512 mg, 4 mmol) and Pd dichloride (177 mg, 1 mmol) was mixed together and allowed to stir at 140 °C without solvent until the reaction mixture melted. Then the reaction mixture was stirred for 2 hours at 180 °C. Next, the flask was cooled down to ambient temperature, and the crude product was finely grounded, collected, and washed with MeOH, EtOH, and water. The product was dried under vacuum for 5 hours. Yield: 276 mg, 45 %.

Synthesis of Pd(TNFB)Cl_2.

Pd(TNFB)Cl_2 was prepared by mixing equimolar amounts of Na_2PdCl_4 and 4,4-Trifluoro-1-(2-naphthyl)-1,3-butanedione in final concentration 1 mM in acetone. The reaction was incubated for 24 hours at 25 °C.

Preparation of Pt-Quercetin complex.

The complex was prepared fresh before each experiment as prolonged storage leads to the formation of Pt nanoparticles. 100 μL of 5 mM quercetin solution was diluted in 390 μL of

methanol and mixed with 5 µL of 100 mM K_2PtCl_4 dissolved in H_2O resulting in 1 mM Pt-quercetin (1:1) complex.

Ferritin was isolated according to previous reports.[4]

Catalysts that were prepared according to published work: i) Pd-Spermine[5], ii) palladium(II) phenanthroline diacetate[6], and iii) Pd(dipic)$(TPP)_2$.[7]

Preparation of phenanthroline metal complexes for DNA binding studies.
2 µL of 100 mM 1,10-phenanthroline was mixed in 196 µL of DMF with 2 µL of 100 mM solutions of $Pd(OAc)_2$, $AuCl_3$, K_2PtCl_4, $RhCl_3$, and $RuCl_3$ and incubated at room temperature for 3 days yielding 1 mM complex.

Fluorescence measurements
Spectral measurements were carried out in 96-well plates in a volume of 200 µL in water with 25 µM UF, AllylUE or PropUE alone, or in presence of 100 mM Tris HCl (pH 8.8). Spectrophotometric characterizations were done with Spark 10M (Tecan Austria GmbH, Grödig, Austria), the multiwell plate reader.

Determination of limit of detection of Pd.
A serial dilutions of $Pd(OAc)_2$ in H_2O varying in concentrations ranging from 200 µM to 50 pM were prepared. 20 µL of the Pd^{2+} solution were placed to 96-well plate and 180 µL of a master mix containing 20 % acetonitrile, AllylUE and BH were added. Fluorescence was measured after 20 min incubation at 25 °C with the excitation at 365 nm and emission at 450 nm with various gains. The final concentration of BH was 5 mM and AllylUE 50 µM. All measurements were done in triplicate.

Preparation of quadruplex DNA oligonucleotide.
The oligonucleotide Ag45 (seq. GGGTTAGGGTTAGGGTTAGGGTTAGGGTTAGGGTTAGGGTTAGGG) was dissolved in 100 mM Tris.HCl (pH 7.6), 100 mM KCl and 2 mM $MgCl_2$ in final concentration 50 µM. The solution was heated to 98 °C and allowed to slowly cool down to room temperature.

Preparation of single-strand DNA oligonucleotide.
DNA oligonucleotide (seq. ATATGCTAGCCACCATGCTCTCCGCCCTCGCCCGGCCTGTCAGCGCTGCTCTCCGC CGCAGCTTCAGCACCTCGGCCCAGAACAATGCTAAAGTAGGATC) was diluted into 100 mM Tris.HCl (pH 7.6), 100 mM KCl and 2 mM $MgCl_2$ in final concentration 50 µM and heated to 98 °C for 2 min and immediately placed on ice.

Preparation of double-stranded DNA oligonucleotide.
25 µL of each complementary 100 µM oligonucleotides (seq. CTAGAGGGACACAATGGACGAGTCTAGAGCGGGTACCTGTGCTGCAAATCTCCC GTCATTTAACGGCCGACATGAGAGA, and AGCTTCTCTCATGTCGGCCGTTAAATGACGGGAGATTTGCAGCACAGGTACCCGC TCTAGACTCGTCCATTGTGTCCCT) and 1.5 µL of 5 M NaCl were heated to 98 °C and allowed to slowly cool down.

Preparation of Pd-quadruplex DNA complexes.

The Ag45 was mixed with Pd catalysts in 100 mM Tris.HCl (pH 7.6), 100 mM KCl and 2 mM $MgCl_2$ with a final concentration of 10 μM (Ag45) and 100 μM (Pd) and incubated 30 min at room temperature. The reaction volume was 10 μL and all catalysts (with the exception of Na_2PdCl_4) were used as 10× concentrated 1 mM solution in DMF, therefore the final reaction mix contains 10 % DMF.

Preparation of metal-DNA complexes.

The DNA oligonucleotide diluted in 100 mM Tris.HCl (pH 7.6), 100 mM KCl and 2 mM $MgCl_2$. 10 μM DNA was incubated with tested complexes, 100 μM each for 30 min.

Preparation of Pd-protein complexes.

20 μg of *Pyrococcus furiosus* (Pfu)ferritin or bovine serum albumin (BSA) were incubated with 100 μM Pd compound for several hours in 150 mM NaCl and 25 mM hemisodium HEPES.

References:

(1) Kofoed, J.; Darbre, T.; Reymond, J. L. *Org. Biomol. Chem.* **2006**, *4*, 3268.

(2) Miller, M. A.; Askevold, B.; Mikula, H.; Kohler, R. H.; Pirovich, D.; Weissleder, R. *Nat. Commun.* **2017**, *8*, 15906.

(3) Lokesh, K. S.; Adriaens, A. *Dyes Pigm.* **2013**, *96*, 269.

(4) Pekarik, V.; Peskova, M.; Guran, R.; Novacek, J.; Heger, Z.; Tripsianes, K.; Kumar, J.; Adam, V. *Dalton Trans.* **2017**, *46*, 13690.

(5) Navarroranninger, C.; Perez, J. M.; Zamora, F.; Gonzalez, V. M.; Masaguer, J. R.; Alonso, C. *J. Inorg. Biochem.* **1993**, *52*, 37.

(6) Milani, B.; Alessio, E.; Mestroni, G.; Sommazzi, A.; Garbassi, F.; Zangrando, E.; Brescianipahor, N.; Randaccio, L. *J. Chem. Soc.-Dalton Trans.* **1994**, 1903.

(7) Espinet, P.; Miguel, J. A.; GarciaGranda, S.; Miguel, D. *Inorg. Chem.* **1996**, *35*, 2287.

SUPPLEMENTARY FIGURES:

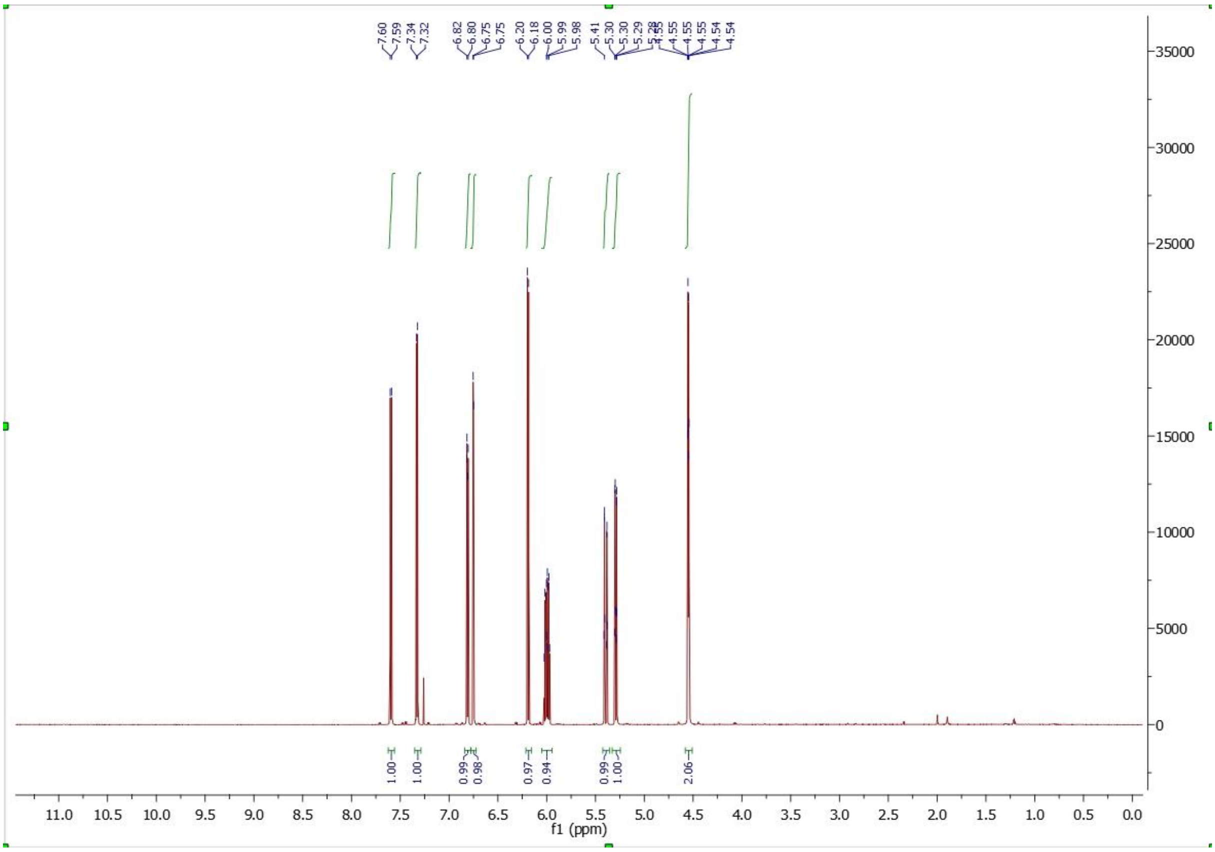

Figure S1. ^{1}H-NMR spectra of AllylUE probe.

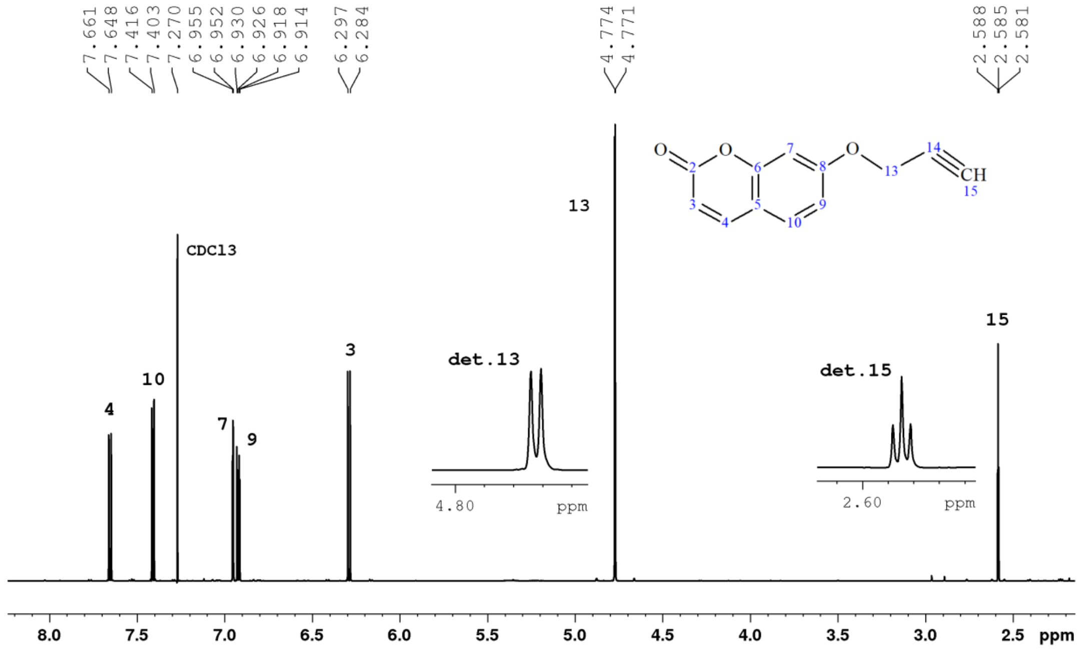

Figure S2. ^{1}H-NMR spectra of PropUE probe.

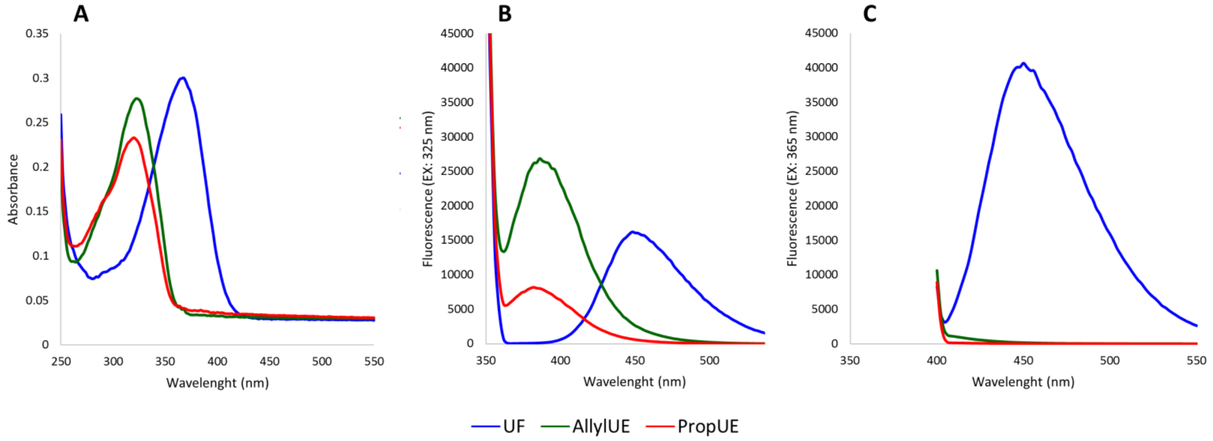

Fig. S3. Spectral characterizations of UF, **1** (AllylUE), and **2** (PropargylUE). Absorption spectra (**A**) and fluorescence spectra with excitation at 325 nm (**B**) and 365 nm (**C**) were recorded in 100 mM Tris HCl (pH 8.8). The fluorescence spectra were acquired with gain 45. The UF absorption maximum of 325 nm in aqueous neutral or acidic solutions shifts to 365 nm at basic pH due to hydroxyl group deprotonation. Regardless of pH, **1** and **2** absorb at 325 nm due to inability to produce deprotonated species (**A**). Excitation at 325 nm induces a fluorescence response of both probes at λ_{em} 385 nm and a fluorescence of UF at λ_{em} 450 nm (**1**). The excitation at 365 nm induces strong blue emission of the deprotonated UF at λ_{em} 450

nm while **1** and **2** are virtually emissionless (**C**). Quantitation of fluorescence emission at 450 nm with excitation at 365 nm provides excellent sensitivity in the determination of uncaged UF with up to 250-fold fluorescence increase.

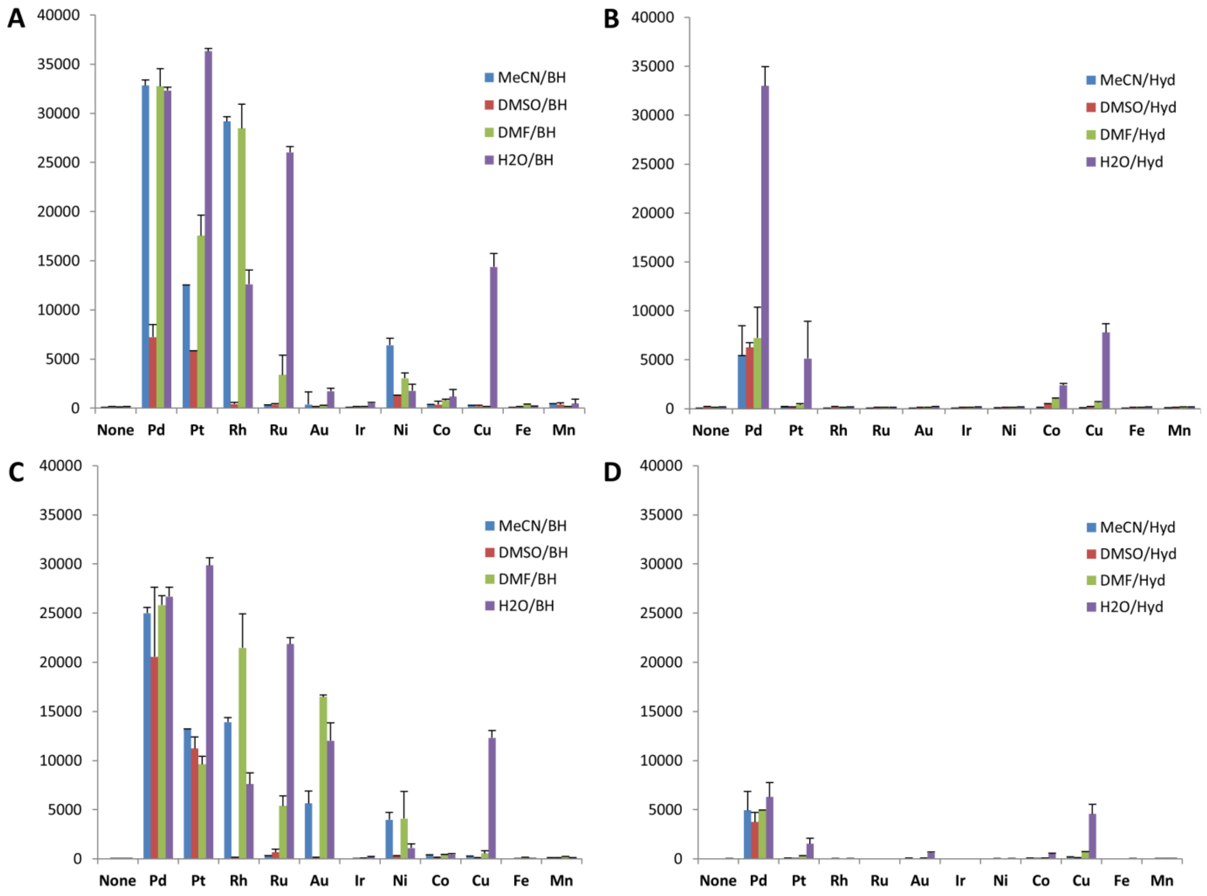

Figure S4. Quantitative determination of metal specificity of AllylUE (**A**, **B**) and PropUE (**C**, **D**) in various solvents in presence of BH (**A**, **C**) and hydrazine (**B**, **D**) as reducing agents. Pd = $Pd(OAc)_2$ =, Pt = K_2PtCl_4, Rh = $RhCl_3$, Ru = $RuCl_3$, Au = $AuCl_3$, Ir = K_3IrCl_6, Ni = $Ni(OAc)_2$, Co = $CoCl_2$, Cu = $CuSO_4$, Fe = $FeCl_3$, Mn = $MnCl_2$, Hyd = hydrazine hydrate (10 mM), BH = sodium borohydride (5 mM), MeCN = acetonitrile (20 %), DMSO = dimethylsulfoxide (20 %), DMF = dimethylformamide (20 %). The total reaction volume was 200 µL with 25 µM AllylUE or PropUE (y-axis represents fluorescence intensities).

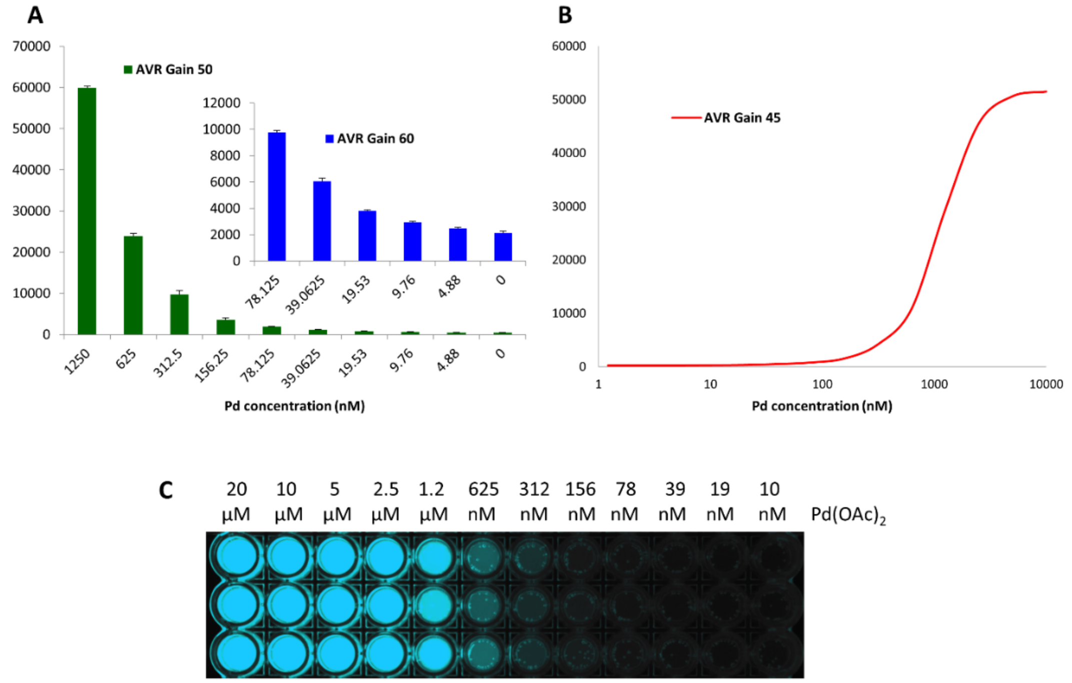

Figure S5. Detection limit for $Pd(OAc)_2$ in 20 % MeCN in presence of 5 mM BH and 50 µM AllylUE. The fluorescence measurements (Excitation: 365 nm, Emission: 450 nm) with different gains (**A**) for selected Pd concentrations (y-axis represents fluorescence intensities). The results for all dilutions at gain 45 showing system saturation level (**B**). The image of 96-well plate under UV transilluminator in triplicate (**C**). Each raw represents individual replica.

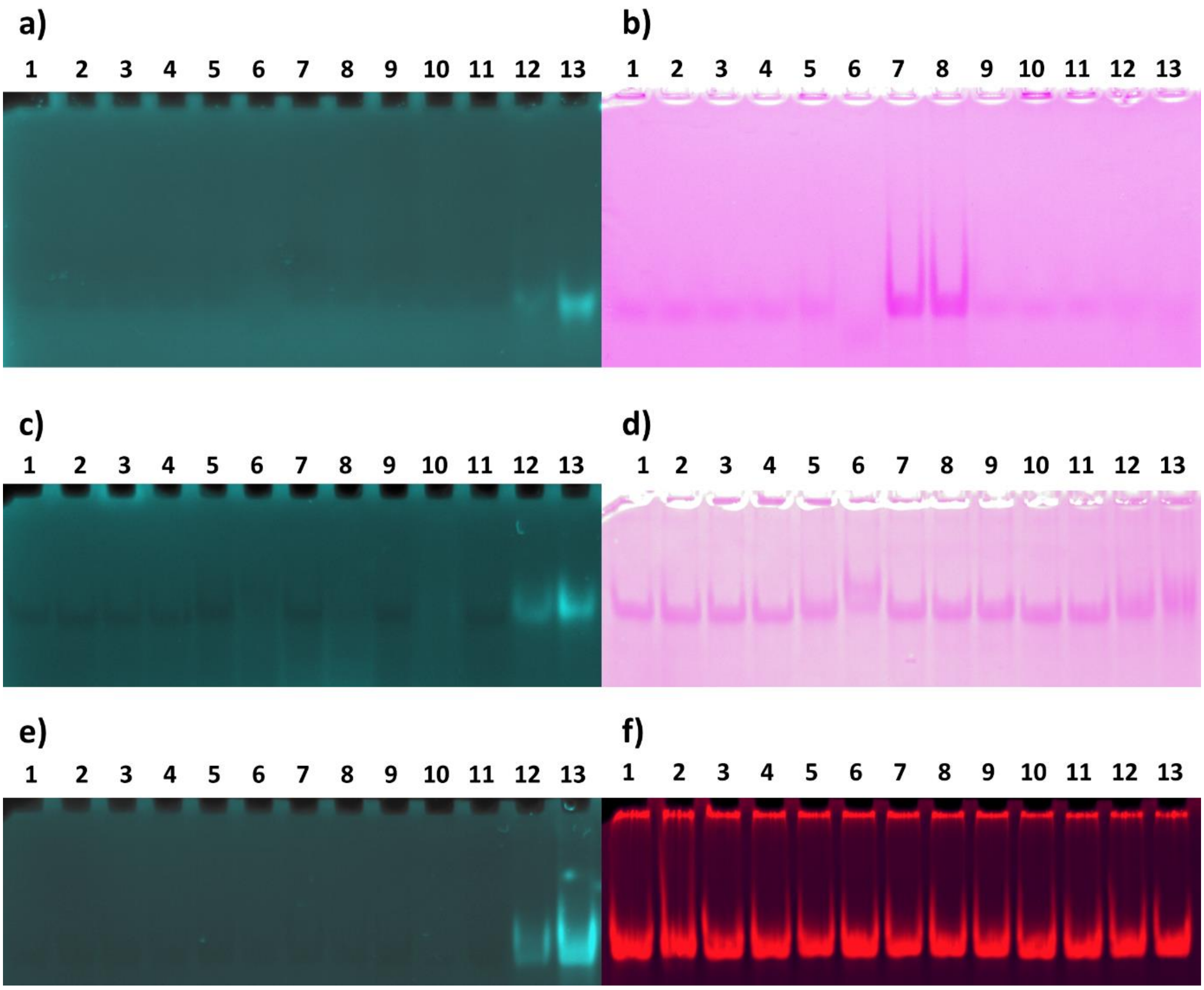

Figure S6. Detection od DNA complexes with Ru and Pt complexes through activation of UPE (a, c, e). Types of DNA analysed were quadruplex DNA (a, b), single stranded DNA (c, d) and double stranded DNA (e, f). After UPE activation, the gels were stained with crystal violet (b, d) and ethidium bromide (f) in order to identify position of the DNA in the gel. The complexes were resolved in 16 % PAG in Tris-Borate buffer. The used catalysts were: 1 - None, 2 – cisplatin, 3 – carboplatin, 4 – oxaliplatin, 5 – Ru phenanthroline, 6 – Pt phenanthroline, 7 – Ru quercetin, 8 – Pt quercetin, 9 - Chloro(pentamethylcyclopentadienyl)(cyclooctadiene)ruthenium(II), 10 - K_2PtCl_4, 11 - $RuCl_3$, 12 - Pd-BBDPPE, 13 - Pd(dipic)$(TPP)_2$
